# Vogt-Koyanagi-Harada disease following influenza vaccination

**DOI:** 10.1016/j.ajoc.2022.101516

**Published:** 2022-04-10

**Authors:** Fahmeeda Murtaza, Austin Pereira, Mark S. Mandelcorn, Alexander J. Kaplan

**Affiliations:** a Temerty Faculty of Medicine, University of Toronto, Toronto, Ontario, Canada; b Department of Ophthalmology and Vision Sciences, University of Toronto, Toronto, Ontario, Canada; c Toronto Western Hospital, University of Toronto, Toronto, Ontario, Canada

**Keywords:** Vogt-Koyanagi-Harada, VKH, Uveitis, Influenza vaccination, Vaccine, HLADR4

## Abstract

**Purpose:**

To report a case of Vogt–Koyanagi–Harada (VKH) disease following influenza vaccination.

**Observations:**

A 30-year-old Filipino male developed bilateral pain, redness, photophobia, floaters, headache and tinnitus 2 days after receiving the annual influenza vaccine. He presented to the emergency department 5 days after symptom onset. His past medical and ocular history was unremarkable. His best-corrected distance visual acuity (BCVA) was 20/100 in the right eye (OD) and 20/150 in the left eye (OS). Slit-lamp examination revealed fine keratic precipitates and 1+ anterior chamber cells in both eyes (OU). Dilated fundus examination revealed 1+ vitreous cells with trace haze and multiple serous retinal detachments OU. Magnetic resonance imaging (MRI) of the brain and chest X-ray were unremarkable. Serologic testing was negative for infectious, inflammatory and neoplastic causes. The patient tested positive for HLA-DR4. A diagnosis of acute Vogt-Koyanagi-Harada disease was made and high-dose oral prednisone, intravitreal triamcinolone acetonide and mycophenolate mofetil were needed to achieve quiescence. At 6 months follow-up, our patient remains in remission with no active intraocular inflammation or subretinal fluid. His BCVA has improved to 20/50 OD and 20/30 OS.

**Conclusion and importance:**

The annual influenza vaccine may be a trigger for onset or recurrence of VKH in genetically susceptible individuals.

## 1 Introduction

Vogt-Koyanagi-Harada (VKH) disease is a rare, granulomatous inflammatory disorder targeting melanocyte-rich tissues of the uvea, meninges, skin and hair. Ocular manifestations include bilateral panuveitis, serous retinal detachments, and optic disc hyperemia. The disease is divided into four consecutive stages: prodromal, acute uveitic, convalescent, and chronic recurrent, which have been extensively described in the literature.^1^ The pathogenesis of VKH is thought to be a T-cell mediated auto-immune reaction to antigens found on melanocytes.^2^ VKH is strongly associated with the HLA-DR4/HLA-DRB1*04 alleles, although this association varies across different ethnic groups.^2^ Left untreated, VKH can lead to complications such as glaucoma, cataract, choroidal neovascularization, or retinal atrophy.^3^ Only a few cases of VKH following vaccinations have been reported worldwide,^4^, ^5^, ^6^, ^7^, ^8^, ^9^, ^10^, ^11^, ^12^, ^13^, ^14^ and this report seeks to add to the literature on this subject.

## 2 Case presentation

**Fig. 1:**
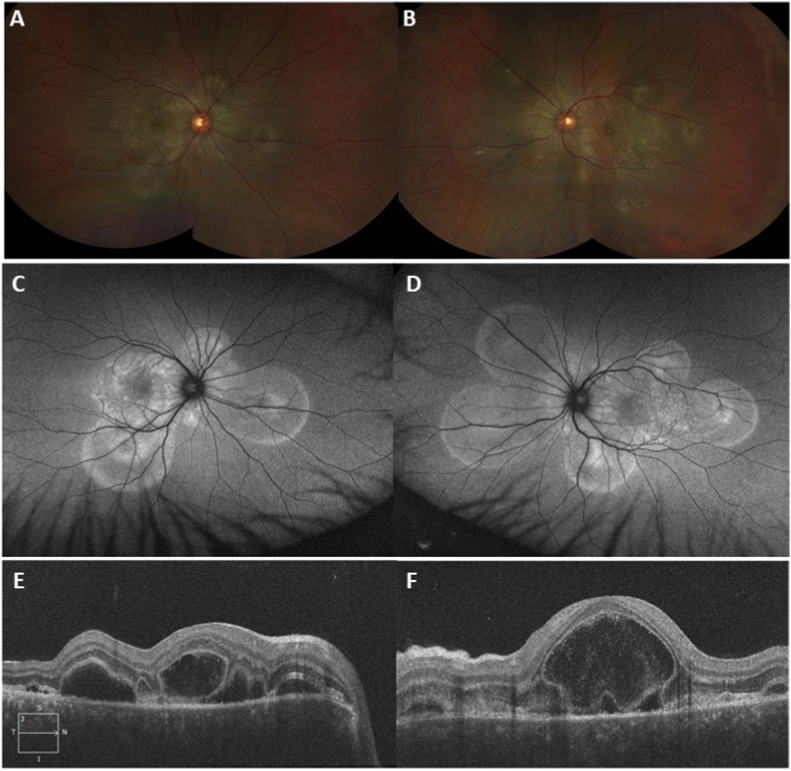
Fundus photos showing bilateral serous retinal detachments in the right (A) and left (B) eye and fundus auto-fluorescence images demonstrating hyper-autofluorescence over areas of serous retinal detachments in the right (C) and left (D) eye. Spectral-domain optical coherence tomography images demonstrating multiple pockets of subretinal and intraretinal fluid with secondary choroidal thickening in the right (E) and left (F) eye.

**Fig. 2:**
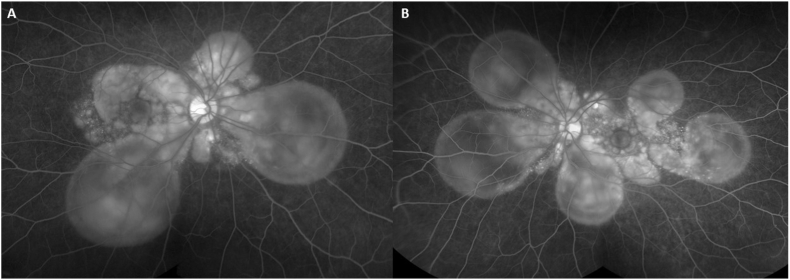
Late frame fluorescein angiography of the (A) right and (B) left eye 2 weeks following the influenza vaccine demonstrating multiple pinpoint hyperfluorescent spots pooling into subretinal space.

A 30-year-old Filipino male was referred to a tertiary ophthalmology clinic with complaints of bilateral pain, redness, photophobia, floaters, headache, tinnitus, and neck stiffness that started 2 days after receiving an intramuscular quadrivalent inactivated influenza vaccine. He presented to the emergency department 5 days after symptom onset. The patient denied any history of ocular trauma or surgery. A computed tomography (CT) scan of the brain completed in the emergency room was unremarkable. His best-corrected distance visual acuity (BCVA) was 20/100 in the right eye (OD) and 20/150 in the left eye (OS). Intraocular pressures were 15 OD and 18 OS. Slit-lamp examination revealed fine keratic precipitates and 1+ cells in the anterior chamber in both eyes (OU). Dilated fundus examination revealed bilateral multifocal serous retinal detachments corresponding to pinpoint hyperfluorescence on fluorescein angiography demonstrated in Fig. 1, Fig. 2. MRI of the brain was within normal limits. Chest X-ray and laboratory workup including CBC, ESR, CRP, Creatinine, ANA, ACE, proteinase 3, myeloperoxidase, syphilis IgG, VDRL and Quantifon GOLD were all negative. HLA testing was positive for HLA-DR4.

The clinical presentation was consistent with acute onset Vogt-Koyanagi-Harada disease. The patient denied any prior history of uveitis but reported experiencing a one-week episode of blurry vision after receiving the influenza vaccine seven years ago, which self-resolved. He did not receive any other influenza vaccines since that time.

Systemic steroid therapy was promptly initiated with 100 mg of oral prednisone for three days, followed by 1 mg/kg of oral prednisone slowly tapered over 3 months to prevent recurrence. Unfortunately, subretinal fluid worsened at 50 mg of prednisone. The prednisone dose was then increased to 60 mg and 3 mg of intravitreal triamcinolone acetonide was also administered. Mycophenolate mofetil was initiated as a steroid-sparing agent. One week following the intravitreal triamcinolone acetonide injection, there was complete resolution of subretinal fluid. Four weeks later, there was a mild rise in intraocular pressures OU, which were adequately controlled by brimonidine tartrate 0.2%/timolol 0.5%. Headaches, tinnitus, and neck stiffness gradually resolved six weeks after initiation of systemic steroid therapy.

**Fig. 3:**
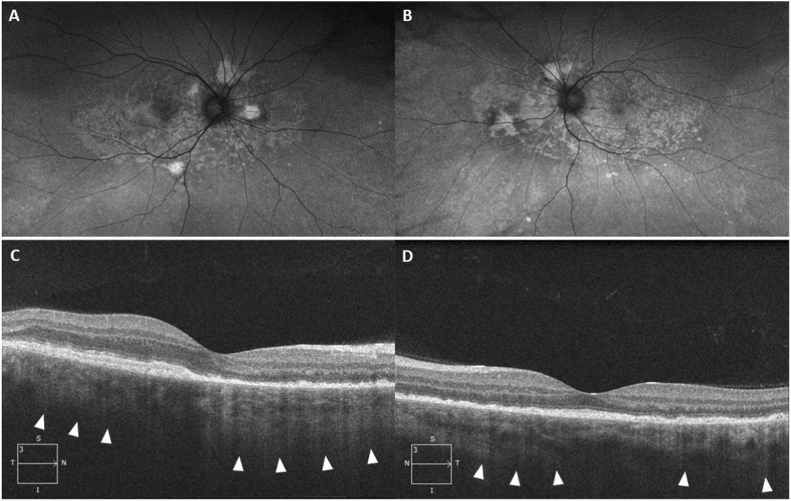
Fundus autofluorescence images reveal multiple punctate hyper and hypo autofluorescent spots correlating to areas of outer retinal atrophy in the right (A) and left (B) eye. Spectral-domain optical coherence tomography images show resolution of subretinal fluid and a transmission defect from outer retinal atrophy (white arrows) in the right (C) and left (D) eye.

The patient remained in remission at 6 months follow-up, with no evidence of cellular inflammation and resolution of subretinal fluid (Fig. 3). A sunset glow fundus from choroidal depigmentation could be appreciated. His best corrected visual acuity improved to 20/50 and 20/30 in the right and left eye, respectively.

## 3 Discussion

We report the first case in the literature of Vogt-Koyanagi-Harada disease suspected to have developed after influenza vaccination in an HLA-DR4 positive patient. Our patient previously experienced a similar episode of blurred vision following influenza vaccination seven years ago, suggesting that he may have been primed to vaccine peptides after this dose and reactivated following the recent dose. The time difference between vaccination and onset of uveitic symptoms strongly suggests that our patient may have developed VKH from the influenza vaccine.

To date, there are two reports of VKH suspected to have developed following influenza vaccination.^,6^ In 2016, Dr. Moosang Kim^6^ described a case of a 52-year-old female that developed bilateral red eyes, reduced vision and serous retinal detachments 1 month following influenza vaccination. Infectious markers were negative. Her HLA-DR4 status was not reported. In 2009, Gallagher and colleagues^4^ reported a case of a 44-year-old female that developed bilateral red eyes, reduced vision and pain 1 month following influenza vaccination. Given the considerable time difference between vaccination and onset of symptoms, it unlikely that VKH developed secondary to influenza vaccination in these cases. Ocular manifestations of VKH typically emerge during the acute uveitic stage, which occurs within two weeks of disease onset.^1^ Furthermore, past reports of uveitis associated with influenza vaccination indicate a median time period of 1 day between vaccination and symptom onset.^15^ The brief time period of 2 days between vaccination and symptom onset in our patient strongly suggests that the VKH developed secondary to the influenza vaccine.

Only a few cases of VKH following vaccinations have been documented globally, namely after the yellow fever,^8^^,^^9^ Bacillus Calmette–Guérin,^7^ hepatitis B virus (HBV),^9^ and SARS-CoV-2^10^, ^11^, ^12^, ^13^, ^14^ vaccines. These reports suggest a short time interval of less than two weeks between vaccination and onset of symptoms, which is in keeping with our patient's presentation. The majority of these cases were reported following administration of the SARS-CoV-2 vaccines, likely from the recent widespread administration across the globe. In most cases, inflammation was well-controlled with only oral corticosteroids, resulting in excellent visual outcomes within 6 months of treatment initiation. Interestingly, none of these cases involved a genetically susceptible individual, as was the case with our patient.

Generally, uveitis is a rare adverse event of vaccinations, with an incidence of about 10.5 in 100,000.^16^ To date, over 300 cases of post-vaccination uveitis have been reported.^15^^,^^17^ In 2016, Benage and Fraunfelder^15^ identified 289 such cases using Medline literature searches and a systematic review of three surveillance systems. They found that 9.7% of identified cases were caused by the influenza vaccine, making it the third most common vaccine to cause uveitis behind the HBV (40.5%) and human papillomavirus (15.6%) vaccines. The majority of these cases were in young (mean age 30 years), females (72.1%), with a short time interval (median 16 days) between vaccination and symptom onset. These cases were commonly anterior, mild and transient, that responded promptly to topical corticosteroids and had good final visual outcomes.^15^^,^^17^

The exact mechanism for development of VKH, or uveitis, post-vaccination remains unclear. In genetically susceptible individuals, VKH is caused by a TH-1 mediated reaction to antigens found on melanocytes, following a viral trigger.^2^ Shi and colleagues^2^ confirmed the association between VKH and HLA-DR4/DRB1*04 alleles, identifying HLA-DRB1*0404, 0405, and 0410 as risk sub-alleles and 0401 as a protective sub-allele for VKH. A few immunological factors are believed to play a role in the development of uveitis following vaccination, including molecular mimicry between vaccine peptide fragments and uveal self-peptides, a delayed-type hypersensitivity reaction with deposition of immune complexes, and an immune reaction to adjuvants.^18^, ^19^, ^20^ It has been proposed that in live, attenuated vaccines, inflammation may be triggered directed by the viral peptides,^19^ while in inactivated or subunit/conjugate vaccines, inflammation may be caused by adjuvants, such as aluminum salts.^20^

## 4 Conclusion

To the best of our knowledge, this is the first case of Vogt-Koyanagi-Harada disease suspected to have developed following the influenza vaccine in an HLA-DR4 positive patient. We encourage ophthalmologists and uveitis specialists to consider the influenza vaccine, as well as other vaccines, as a possible cause for the onset or trigger for recurrence of VKH disease, especially in genetically susceptible individuals. Further research is required to elucidate the mechanism by which vaccines activate or trigger VKH disease, and more generally, uveitis.

## Patient consent

Toronto Western Hospital and the University Health Network have waived the need for REB review of case reports. Verbal consent to publish the case report was obtained. This report does not contain any personal information that could lead to the identification of the patient.

## Funding

This work was not supported by any funding from agencies in the public, commercial, or not-for-profit sectors.

## Authorship

All authors attest that they meet the current ICMJE criteria for Authorship.

**Fahmeeda Murtaza:** Conceptualization, Visualization, Writing – Original Draft; **Austin Pereira:** Conceptualization, Visualization, Writing - Review & Editing; **Mark S. Mandelcorn:** Investigation, Writing - Review & Editing; **Alexander J. Kaplan:** Conceptualization, Visualization, Investigation, Writing - Review & Editing.

## Meeting presentations

None.
